# High-dose-rate brachytherapy and hypofractionated external beam radiotherapy combined with long-term hormonal therapy for high-risk and very high-risk prostate cancer: outcomes after 5-year follow-up

**DOI:** 10.1093/jrr/rrt128

**Published:** 2013-11-11

**Authors:** Hiromichi Ishiyama, Takefumi Satoh, Masashi Kitano, Ken-ichi Tabata, Shouko Komori, Masaomi Ikeda, Itaru Soda, Shinji Kurosaka, Akane Sekiguchi, Masaki Kimura, Shogo Kawakami, Masatsugu Iwamura, Kazushige Hayakawa

**Affiliations:** 1 Department of Radiology and Radiation Oncology, Kitasato University School of Medicine, 1-15-1 Kitasato, Sagamihara 252-0329, Japan; 2 Department of Urology, Kitasato University School of Medicine, 1-15-1 Kitasato, Sagamihara 252-0329, Japan; 3 Department of Radiology, National Hospital Organization Sagamihara National Hospital , 18-1 Sakuradai, Sagamihara 252-0392, Japan

**Keywords:** high-dose-rate brachytherapy, prostate cancer, androgen deprivation therapy, high-risk, very high-risk

## Abstract

The purpose of this study was to report the outcomes of high-dose-rate (HDR) brachytherapy and hypofractionated external beam radiotherapy (EBRT) combined with long-term androgen deprivation therapy (ADT) for National Comprehensive Cancer Network (NCCN) criteria-defined high-risk (HR) and very high-risk (VHR) prostate cancer. Data from 178 HR (*n* = 96, 54%) and VHR (*n* = 82, 46%) prostate cancer patients who underwent ^192^Ir-HDR brachytherapy and hypofractionated EBRT with long-term ADT between 2003 and 2008 were retrospectively analyzed. The mean dose to 90% of the planning target volume was 6.3 Gy/fraction of HDR brachytherapy. After five fractions of HDR treatment, EBRT with 10 fractions of 3 Gy was administered. All patients initially underwent ≥6 months of neoadjuvant ADT, and adjuvant ADT was continued for 36 months after EBRT. The median follow-up was 61 months (range, 25–94 months) from the start of radiotherapy. The 5-year biochemical non-evidence of disease, freedom from clinical failure and overall survival rates were 90.6% (HR, 97.8%; VHR, 81.9%), 95.2% (HR, 97.7%; VHR, 92.1%), and 96.9% (HR, 100%; VHR, 93.3%), respectively. The highest Radiation Therapy Oncology Group-defined late genitourinary toxicities were Grade 2 in 7.3% of patients and Grade 3 in 9.6%. The highest late gastrointestinal toxicities were Grade 2 in 2.8% of patients and Grade 3 in 0%. Although the 5-year outcome of this tri-modality approach seems favorable, further follow-up is necessary to validate clinical and survival advantages of this intensive approach compared with the standard EBRT approach.

## INTRODUCTION

In the field of radiation oncology, the current strategy for treating high-risk prostate cancer is to combine external beam radiation therapy (EBRT) with long-term androgen deprivation therapy (ADT). Randomized trials have shown not only an improved biochemical control rate, but also an improved overall survival rate (OS) with this combination compared with radiation alone [1, 2] or hormonal therapy alone [3]. Radiation dose escalation without ADT is also well established using 3D conformal beam radiotherapy, intensity-modulated radiotherapy, low- or high-dose-rate (HDR) brachytherapy, and particle beams. Although the combination of dose escalation and ADT has been suggested to offer improved treatment results, little information has been accumulated regarding this approach. Few reports appear to have described the combination of HDR brachytherapy and long-term ADT for high-risk prostate cancer patients.

Among modern radiotherapeutic techniques, brachytherapy is expected to provide an effective approach for delivering radiation doses more safely and precisely compared with 3D conformal radiotherapy or intensity-modulated radiotherapy [4]. In addition, hypofractionated radiotherapy may prove advantageous for treating prostate cancer when compared with other types of cancer, because of the low α:β ratio [5]. We have been treating prostate cancer patients with HDR brachytherapy combined with hypofractionated EBRT using a fractional dose of 3 Gy administered five times per week [6].

The purpose of this study was to report the long-term outcomes of HDR brachytherapy combined with hypofractionated EBRT with long-term ADT for localized prostate cancer.

## MATERIALS AND METHODS

### Patients

The institutional review board approved this retrospective study. A total of 200 consecutive patients with National Comprehensive Cancer Network (NCCN) criteria-defined high-risk (HR) and very high-risk (VHR) prostate cancer were treated using HDR brachytherapy between December 2003 and January 2008. Clinical Stage T3a, a Gleason score of 8–10, and a prostate-specific antigen (PSA) level >20 ng/ml were defined as HR factors. Clinical stage T3b–T4 was defined as the VHR factor. Patients with a single HR factor were classified as HR, and patients with at least the single VHR factor or two HR factors were classified as VHR. Pretreatment evaluation included clinical history, physical examination, blood laboratory findings, pelvic computed tomography (CT), and a bone scan. Magnetic resonance imaging (MRI) was recommended on request. No lymph node dissection was performed. Patients with positive lymph nodes or distant metastasis were excluded. International Prostate Symptom Score (IPSS), previous transurethral resection, and prostate volume were not considered in the selection criteria.

Of the 200 patients, 6 failed to complete the scheduled protocol, and another 16 were lost to follow-up. Analyses were thus performed for 178 of the 200 patients. Characteristics of these 178 patients are shown in Table 1.

**Table 1.** Patients' characteristics

	High–risk	Very–high–risk
n	96	82
Age	72 (57–87)	72 (54–87)
Clinical T stage		
T1c	38	4
T2a	3	1
T2b	26	3
T2c	8	1
T3a	20	43
T3b	1	28
T4	0	2
Gleason score		
10	2	1
9	8	21
8	21	18
4 + 3	20	23
3 + 4	26	15
6	14	4
5	5	0
Initial PSA (ng/ml)	19.9 (3.3–159)	40.5 (2.7–337.6)
Prostate volume (ml)	20.7 (9.5–63.5)	18.8 (6.9–60.8)
Neoadjuvant ADT (months)	12 (8–28)	13 (7–74)

Values are number or median (range)

PSA, prostate specific antigen; LNI, lymph-node involvement

ADT; androgen deprivation therapy

### Radiotherapy and hormonal therapy

All patients initially underwent ≥6 months (mean, 14 months; median, 12 months; range, 7–74 months) of neoadjuvant ADT, and adjuvant ADT was continued for 36 months after completion of radiotherapy. Neoadjuvant ADT comprised combined androgen blockade with monthly gonadotropin-releasing hormone agonist (GnRHa) injections and 125 mg of flutamide twice daily. Adjuvant ADT consisted of monthly injections of GnRHa. Briefly, patients in the operating room were placed in a lithotomy position under epidural anesthesia. Treatment was initiated using placement of a closed transperineal hollow needle under transrectal ultrasound guidance. Multiple 20- to 25-cm-long, closed-end, 15-G plastic hollow needles were inserted transperineally using a Syed-Neblett plastic template (Alpha-Omega Services, Bellflower, CA). Routinely, 18 needles were implanted. Twelve needles were inserted in the peripheral portion and six needles were inserted in the central portion of the prostate. Flexible cystoscopy was conducted to check that the urethra had not been penetrated by the implanted tubes. The needle tips were left within the urinary bladder, 1.5 cm above the sonographically or cystoscopically defined base of the prostate. Metallic marker seeds were placed transperineally into the base and apex.

After all of these procedures had been completed, the patient underwent CT to obtain scans at 5-mm intervals for CT-based planning. Contours of the planning target volume (PTV), urethra and rectum were outlined according to transverse CT images. The PTV was defined as the prostate gland with or without proximal seminal vesicles, with an additional 3- to 5-mm margin all around. Reference points for the urethra were set on the center of the urethral catheter, and those for the rectal wall were set 5 mm behind the edge of the anterior rectal wall on transverse CT images with 10-mm intervals. Reference points for the PTV were automatically distributed on the surface of the PTV. Dose limitation was set as 10 Gy/fraction for urethral reference points and 4 Gy/fraction for rectal reference points, and we attempted to prescribe 7.5 Gy/fraction to reference points of the PTV (unless the dose limitation was violated) using inverse planning and geometric optimization. Because of urethral and rectal dose limitations, covering the periphery of the PTV with the prescribed dose was difficult in some patients. The mean dose to 90% of the PTV (D90), the prostate volume receiving at least 6 Gy (V6), and the prostate volume receiving at least 9 Gy (V9) were 6.3 ± 0.6 Gy, 91 ± 5%, and 53 ± 9% per fraction, respectively. Five fractions of HDR treatment were administered. After CT-based planning using a Nucletron planning system (Veenendaal, the Netherlands), the first treatment session of HDR brachytherapy was conducted using the Nucletron microSelectron HDR ^192^Ir remote afterloading system. Dwell positions were activated at 5-mm intervals along each catheter. The deepest dwell position could be set at 6.5 mm from the catheter tip if needed. Catheter positions were checked by fluoroscopy before every treatment session and corrected if interfraction needle movements >5 mm were noted. The first treatment session was conducted on the day of implantation, with the subsequent four treatment sessions administered twice daily with an interval of at least 6 h between fractions. Treatment duration was thus 3 days. At 6 days after completion of HDR brachytherapy, patients received EBRT using a dynamic-arc conformal technique, administered with high-energy photons comprising 10-MV X-rays to a total dose of 30 Gy. Total dose was administered in five weekly fraction doses of 3 Gy. The radiation field was limited to the prostate gland with or without proximal seminal vesicles with a 7-mm leaf margin using multileaf collimators.

### Follow-up

Duration of follow-up was calculated from the start of HDR brachytherapy. Toxicities were evaluated using the Radiation Therapy Oncology Group scale [7] at every visit, and all patients were followed up at 3-month intervals during the first year and at 3- to 6-month intervals thereafter. Acute toxicity was defined as toxicity occurring ≤3 months after implantation and late toxicity as that occurring after >3 months. Median follow-up for all patients was 61 months (range, 25–94 months). Biochemical failure was defined according to the Phoenix definition [8]. Biochemical non-evidence of disease rate (bNED) was calculated for all living patients and reflected biochemical failures. Freedom from clinical failure rate (FFcF) was calculated for all living patients and reflected all clinical events (local, regional or distant failure) and salvage ADT. OS reflected all deaths, cancer-related or otherwise.

### Statistical analysis

Univariate analysis (log-rank) was used to examine the predictive value of patient-related factors (clinical T stage (≤T2c vs ≥T3a), Gleason score (≤7 vs ≥8), initial PSA (≤20 ng/ml vs >20 ng/ml), NCCN criteria (HR vs VHR), prostate volume (≤20 ml vs >20 ml), age (≤70 years vs >70 years)) and treatment-related factors (D90 (≤6.3 Gy/fraction vs >6.3 Gy/fraction) and duration of neoadjuvant ADT (<12 months vs ≥12 months)). To evaluate interactions and independent influences on factors, multivariate analysis was performed using Cox regression analysis. Differences were regarded as statistically significant at the *P* < 0.05 level.

## RESULTS

### Efficacy

**Fig. 1.**
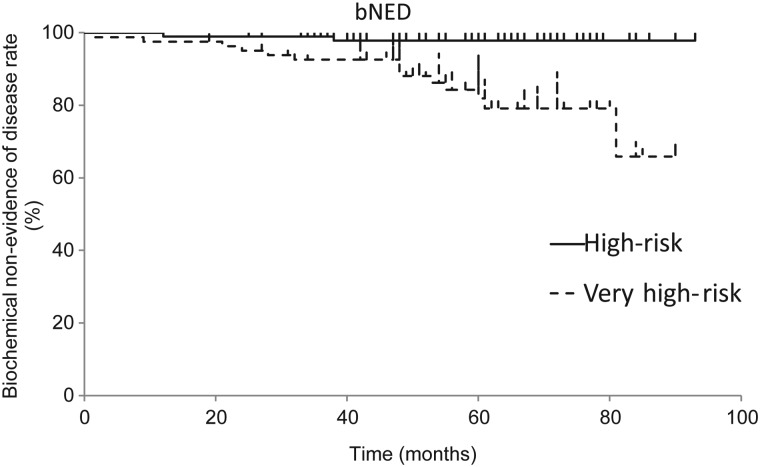
Biochemical non-evidence of disease rates (bNED) for high-risk and very high-risk patients.

The 3- and 5-year bNED were 96.0% (HR, 98.9%; VHR, 92.6%) and 90.6% (HR, 97.8%; VHR, 81.9%), respectively (Fig. 1). The corresponding values for FFcF were 97.4% (HR, 99.0%; VHR, 96.3%) and 95.2% (HR, 97.7%; VHR, 92.1%), respectively. The 3- and 5-year OS rates were 97.7% (HR, 100%; VHR, 95.1%) and 96.9% (HR, 100%; VHR, 93.3%), respectively. Nine patients experienced clinical progression, including 4 patients with bone metastasis, 1 patient with lung metastasis, 1 patient with distant lymph-node metastasis, 1 patient with regional lymph-node metastasis, 1 patient with positive biopsy, and 1 patient who underwent salvage ADT. Five patients died during follow-up including 1 patient who died of interstitial pneumonitis, 1 patient who died of bladder cancer, 2 patients who died of prostate cancer, and 1 patient who died due to an accident.

### Toxicity

**Fig. 2.**
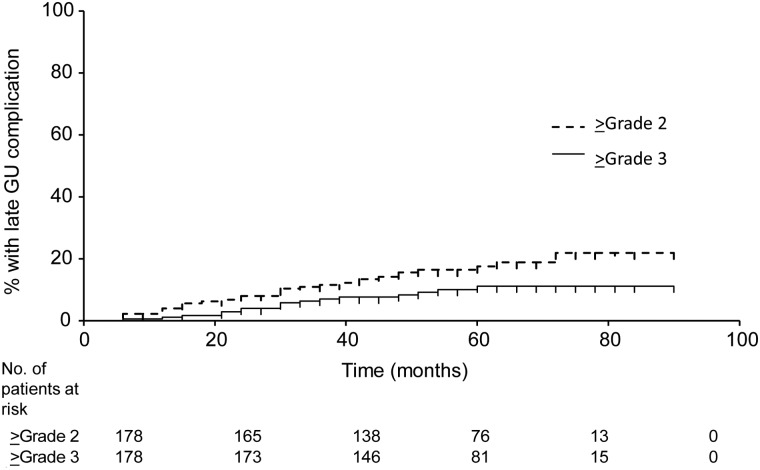
Actuarial incidence of late genitourinary (GU) complications.

The highest Radiation Therapy Oncology Group (RTOG)-defined acute genitourinary (GU) toxicities were Grade 2 in 19 patients (10.7%) and Grade 3 in 10 patients (5.6%). No patients experienced ≥Grade 2 acute gastrointestinal (GI) toxicities. The highest RTOG-defined late GU toxicities were Grade 2 in 13 patients (7.3%) and Grade 3 in 17 patients (9.6%). Most Grade 3 GU toxicities were urinary retention or urethral stricture, which were managed successfully by temporary catheterization or internal urethrotomy. The highest late GI toxicities were Grade 2 in 5 patients (2.8%). No patients showed Grade 3 toxicities. No patients experienced acute Grade 4 or 5 toxicity. Actuarial rates of late GU toxicity are shown in Fig. 2.

### Predictive factors for bNED and FFcF

Table 2 shows the results of uni- and multivariate analyses for factors predicting bNED and FFcF. On univariate analysis, clinical T stage, Gleason score and NCCN risk-group were detected as predictive factors for bNED. Clinical T stage, NCCN risk-group and prostate volume were significant predictors for FFcF. On multivariate analysis, however, no risk factor reached the level of statistical significance.

**Table 2.** Univariate and multivariate analysis of biochemical and clinical disease control rates

Variable		bNED				FFcF			
		Univariate		Multivariate		Univariate		Multivariate	
		p	HR	p	HR	p	HR	p	HR
Stage	≤T2c ≥T3a	0.014*	0.234	0.316	2.165	0.020*	0.127	0.187	5.242
Gleason score	≤7 ≥8	0.047*	0.374	0.215	2.003	0.283	0.494		
PSA level (ng/ml)	≤20 >20	0.309	0.562	^−^		0.688	1.308		
NCCN criteria	High–risk Very–high–risk	<0.001*	0.109	0.083	4.842	0.044*	0.229	0.687	1.465
Age (y.o)	≤70 >70	0.043*	2.813	0.058	0.355	0.922	1.068		
Prostate volume (ml)	≤20 >20	0.218	1.937	−		0.021*	7.759	0.094	0.168
D90 (Gy per fraction)	≤6.3 >6.3	0.909	0.945	−		0.631	0.726		
NeoADT duration (months)	≤12 >12	0.403	0.632	−		0.129	0.304		

* statistical significance

bNED, biochemical non–evidence of disease rate; FFcF, freedom from clinical failure rate; HR, hazard ratio;

PSA, prostate specific antigen; ADT, androgen deprivation therapy; NCCN, national comprehansive cancer network

## DISCUSSION

With more than 5 years of follow-up, HDR and hypofractionated EBRT combined with long-term ADT produced encouraging results. Even with VHR patients, a 5-year bNED of 81.9% could be achieved with this combination. Although continued failures will likely occur with additional follow-up, our result was encouraging compared with the reported bNED of HDR brachytherapy for HR prostate cancer (Table 3), which varies within the range of 60–80% at a 5-year median follow-up with or without hormonal therapy[9–25].

**Table 3.** Selected reports of HDR for high–risk localized prostate cancer (n ≥ 100, median follow-up ≥5 years)

Auther	Year	n	HDR	EBRT	Follow-up (median)	OS	bDFS or bNED	Definition	Hx	H × length
Yoshioka	2011	112 (high, 68)	54Gy in 9fx	none	5.4 years	5-year 97%	5-year bNED Low, 85% Int, 93% High, 79%	Phoenix	84%	Median 36 months (2–131 months)
Agoston	2011	100 (high, 61)	10Gy in 1fx	60Gy	61.5 months	5-year 93.3%	7-year bNED Int, 84.2% High, 81.6%	Phoenix	30%	Mean 17.7 months (4–60 months)
Phan	2007	309 (high, 133)	15–26Gy in 3–4fx	36–50.4Gy	59 months	5-year 91%	5-year bNED Low, 98% Int, 90% High, 78%	ASTRO	36%	NA
Hoskin	2012	197 (high, 86)	34–36Gy in 4fx 31.5Gy in 3fx 26Gy in 2fx	none	4.5-5-years	NA	4-year bNED Int, 95% High, 87%	Phoenix	80%	Median 6.3 months (1–40 months)
Hoskin	2012	110 (high, 54)	2 × 8.5Gy	35.75Gy in 13fx	85 months	5-year 88%	bDFS 5-year, 75% 7-year, 66% 10-year, 46%	Phoenix	77%	Low and int, 6 months High, up to 3 years
Martinez	2011	472 (high, NA)	5.5Gy–11.5Gy × 2–3fx	46Gy	8.2 years	NA	10-year bNED 70.6%	Phoenix	51%	<6 months
Khor	2013	344 (high, 141)	19.5Gy in 3 fractions	46Gy	60.5 months	NA	5-year bNED 79.8%	Phoenix	59%	NA
Kaprealian	2012	165 (high, 156)	18Gy in 3fx 19Gy in 2fx	45Gy	105 months 45 months	5-year 92% 97%	5-year bNED 93.5% 87.3%	Phoenix	76% 92%	Mean 9.5 months Mean 19.2 months
Prada	2012	313 (high, 238)	23Gy in 2fx	46Gy	68 months	5-year 92%	10-year bNED Low, 100% Int, 88% High, 91% Very high, 79%	Phoenix	70%	12 months
Prada	2012	252 (high, 252)	23Gy in 2fx	46Gy	74 months	5-year 88%	bNED 5-year, 84% 10-year, 78%	Phoenix	69%	12 months
Kotecha	2012	229	5.5–7.5Gy × 3	45–50.4Gy	61 months	NA	7-year bDFS Low, 95% Int, 90% High, 57%	Phoenix	42%	9 months
Pellizzon	2008	209 (high, 67)	Median 20Gy (16–24Gy) in 4fx	Median 45Gy (36–54Gy)	5.3 years	5-year 95.7%	3.3-year bNED Low, 91.5% Int, 90.2% High, 88.5%	Phoenix	48%	3–6 months
Galalae	2004	611 (high, 359)	3–4Gy × 4fx 8–9Gy × 2fx 5.5–11.5Gy × 2–3fx	45.6–50Gy	mean 5 years	5-year 85%	5-year bNED Low, 96% Int, 88% High, 69%	ASTRO	29%	Median 4 months
Galalae	2006	324 (high, 80)	5.5–6.5Gy × 3fx 8.25–15Gy × 2fx	45.6–50Gy	5.3 years	5-year 90%	5-year bNED Low, 85% Int, 81% High, 69%	ASTRO	0%	NA
Deger	2005	411 (high, 295)	9–10Gy × 2fx	40–50.4Gy	5 years	5-year 87%	5-year bDFS Low, 81% Int, 65% High, 59%	ASTRO	NA	NA
Kalkner	2007	154 (high, 66)	20Gy in 2fx	50Gy	median 6 years	NA	5-year bNED or bDFS Low, 97% Int, 83% High, 83% Very high, 51%	Phoenix	100%	6–9 months
Demanes	2005	209 (high, 47)	22–24Gy in 4fx	36Gy in 20fx	7.25 years	Crude 79%	5-year bDFS Low, 93% Int, 93% High, 83%	Phoenix	0%	NA
Present study		178 (high, 178)	31.5Gy in 5fx	30Gy in 10fx	61 months	5-year 96.6%	5-year bNED 90.6%	Phoenix	100%	≥42 months

EBRT, external radation therapy; bDFS, biochemical dsease free survival rate; bNED, biochmical non–evidence of disease rate; Hx, hormonal therapy; ASTRO, american society of radation oncology OS, overall survival rate

On the other hand, Japanese-specific high-sensitivity to hormonal therapy may have some impacts on our outcomes [26]. Table 4 shows selected Japanese series of high-risk prostate cancer patients treated with conventional EBRT and hormonal therapy [27–31]. These favorable outcomes seem to be equal to our HDR approach. From the data of the 5-year follow up, it seems that our tri-modality therapy did not show any advantages over conventional EBRT with hormonal therapy.

**Table 4.** Selected reports of ERRT for high-risk localized Japanese prostate cancer patients

Auther	Year	n	ERRT	Follow-up (median)	OS	bDFS	Definition	Hx	Hx length
Takaha	2011	75 (High, 100%)	70Gy	59 months	5-year High, 98.3%	5-year High, 87.4%	bDFS Phoenix	100%	median 27months (8–63 months)
Sakamoto	2010	70 (High, 100%)	median 70Gy (60–70Gy)	64.9 months	5-year High, 90.3%	5-year High, 60.5%	bDFS Phoenix	100%	median 4 months (3–16 months)
Nakamura	2008	679 (High, 66.3%)	>60Gy	46 months	5-year 93.0%	5-year Low, 90.8% Int, 75.7%High, 67.6%	bDFS ASTRO	82.90%	Neoadjuvant, median 6months (1–68 months) Adjuvant, median 38 months (1–109 months)
Mitsumori	2006	27 (High, 100%)	70Gy	63 months	5-year High, 83.0%	5-year High, 43.0%	bDFS Phoenix	100%	3 months
Saito	2006	78 (High, 100%)	median 70Gy (60–70Gy)	55 months	5-year High, 94.9%	NA	NA	100%	1–36 months or longer

ERRT, external radiation therapy; bDFS, biochemical disease free survival rate; Hx, hormonal therapy; ASTRO, american society of radiation oncology OS, overall survival rate

Regarding late toxicities, 9.6% of our patients suffered from Grade 3 urethral toxicities after treatment. Meanwhile, the incidence of Grade 3 toxicity after EBRT was very rare [28]. HDR brachytherapy is associated with relatively severe GU toxicities, such as urethral stricture [11, 23, 24, 32]. Although the severity of GU toxicities in our study population was relatively high, all cases were manageable by medical or surgical intervention. Compared with patients (*n* = 298) treated before June 2008 when more strict urethral dose limitation was applied, the patients treated after that month (*n* = 279) show a significantly lower rate of Grade 3 late GU toxicity (11% vs 1%), although follow-up duration for the latter group was immature (54 months vs 30 months). Detailed data about updated DVH analysis is planned for publication in another study.

The biological equivalent dose (BED) for our protocol was a total of 205.7 Gy2 (130.7 Gy2 for D90 of HDR; 75 Gy2 for the isocenter of EBRT) based on α/β = 2. Compared with other institutions [9–15], our total dose was not excessive.

Although the data for ADT toxicities were not available in our study, ADT may lead to numerous toxicities, such as osteoporosis, obesity, sarcopenia, lipid alterations, insulin resistance, and increased risk for diabetes and cardiovascular morbidity [33]. These potential side-effects may reduce quality of life and overall survival. Combining ADT with EBRT is warranted because it will improve overall survival in high-risk patients [1]. However, there has been no randomized trial of the combination of ADT and HDR brachytherapy. Further trial is needed for exploring whether ADT really improves overall survival when it is combined with HDR brachytherapy.

Without whole pelvic irradiation, only 1 patient in our study population showed regional lymph node recurrence. The probability of lymph node involvement based on the Roach equation [34] was >30% for half of our patients. It is generally considered that whole pelvic irradiation may have potential benefit for these HR patients [35]. In our population, however, potential lymph-node metastasis was controlled by ADT alone, without irradiation. Unknown mechanisms and/or tumor-specific immune responses such as cytotoxic T lymphocyte (CTL) activity might be evoked through our protocol, although this remains to be established [36, 37].

Several limitations must be considered when interpreting the results of this study. First, our protocol did not apply a uniform duration of neoadjuvant ADT. Many of our patients had been on waiting lists for >6 months because of our limited capacity; they were treated with ADT while on the waiting list, and half of our patients received neoadjuvant ADT for ≥12 months. Second, the lack of testosterone data may confound the interpretation of the bNED results. After long-term ADT, delays in PSA recovery may lead to delays in biochemical failure. Third, our median follow-up of 61 months may have been insufficient, considering the provision of long-term adjuvant ADT for 36 months. Fourth, the excludion of 22 patients from the analysis (due o failure to complete the scheduled protocol or loss to follow-up) might have led to some degree of selection bias in the present study.

## CONCLUSION

Although the 5-year outcome of this tri-modality approach seems favorable, further follow-up is necessary to validate clinical and survival advantages of this intensive approach compared with the standard EBRT approach.

## FUNDING

This work was supported by JSPS KAKENHI Grant Number 24791334.
